# Subsurface automated samplers for eDNA (SASe) for biological monitoring and research

**DOI:** 10.1016/j.ohx.2021.e00239

**Published:** 2021-10-14

**Authors:** Nathan Formel, Ian C. Enochs, Chris Sinigalliano, Sean R. Anderson, Luke R. Thompson

**Affiliations:** a Ocean Chemistry and Ecosystems Division, Atlantic Oceanographic and Meteorological Laboratory, NOAA, 4301 Rickenbacker Cswy, Miami, FL 33149, USA; b Cooperative Institute for Marine and Atmospheric Studies, University of Miami, 4600 Rickenbacker Cswy, Miami, FL 33149, USA; c Northern Gulf Institute, Mississippi State University, 2 Research Blvd, Starkville, MS 39759, USA

**Keywords:** Water sampler, Automation, Survey, eDNA, Open source, 3D printing

## Abstract

Sampling of environmental DNA (eDNA) in seawater is an increasingly common approach to non-invasively assess marine biodiversity, detect cryptic or invasive species, and monitor specific groups of organisms. Despite this remarkable utility, collection and filtration of eDNA samples in the field still requires considerable time and effort. Recent advancements in automated water samplers have standardized the eDNA collection process, allowing researchers to collect eDNA day or night, sample in locations that are difficult to access, and remove the need for highly trained personnel to perform sampling. However, the high cost of purchasing or building these samplers represents a financial hurdle to widespread application. To overcome this difficulty, we have designed and built a low-cost subsurface automated sampler for eDNA (SASe). Each sampler is submersible to 55 m, can filter a pre-programmable volume of water, and preserves eDNA at the site of collection. SASe samplers have replaceable filters and a low build cost (∼280 USD vs. >100,000 USD for other eDNA samplers), which facilitates repeated field sampling at fine spatial and temporal scales. Lab testing has shown the SASe to be as effective as a standard desktop peristaltic pump for sampling, preserving, and recovering marine eDNA. SASe design files and operating code are open-source, promoting the use of this tool to meet a range of future eDNA research applications, including project-specific customizations to the current design.

## Graphical abstract

**Figure:**
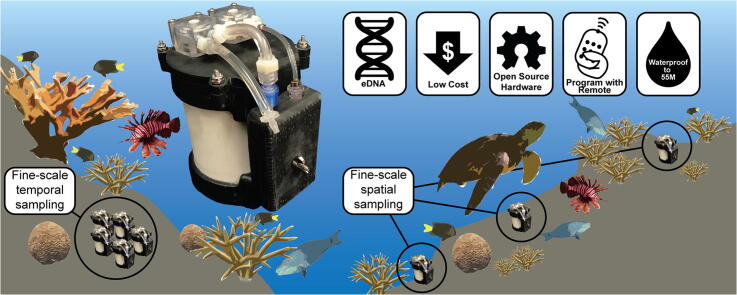

**Specifications table:** 

Hardware name	*Subsurface Automated Sampler for eDNA (SASe)*
Subject area	● Biological Sciences (e.g., Microbiology and Biochemistry) ● Environmental, Planetary and Agricultural Sciences ● Educational Tools and Open Source Alternatives to Existing Infrastructure
Hardware type	● Biological sample handling and preparation ● Field measurements and sensors
Open Source License	Creative Commons Attribution-ShareAlike license
Cost of Hardware	< 280.00 USD
Source File Repository	Mendeley: https://doi.org/10.17632/y3dgh36zt9.2

## Hardware in context

Environmental DNA (eDNA) is the trace DNA left behind by organisms as they interact with their environment [1]. In marine and other aquatic environments, eDNA represents whole bacteria or plankton cells or cellular debris (e.g., tissue, scales, mucous, feces) from larger organisms like invertebrates, fish, and mammals [2], [3]. Following eDNA collection and filtration, downstream techniques can be used to target entire communities of organisms using universal primers (e.g., 12S rRNA) [4], known as amplicon sequencing or metabarcoding. Alternatively, it can be used to target and detect single species of interest using quantitative PCR (qPCR) primers [5].

Collecting water samples for eDNA analysis has proven to be a useful tool for quantifying biodiversity and detecting cryptic and evasive taxa that traditional surveying techniques (e.g., trawls, visual surveys, and microscopy) may otherwise underestimate or miss entirely [6], [7], [8]. Furthermore, eDNA sampling methods are non-invasive and amenable to researchers with a wide range of expertise [5]. Similar to traditional biological surveys, however, there remains logistical and financial obstacles to collecting eDNA samples in the field [9], [10].

To reduce the logistical challenges of eDNA collection, automated eDNA samplers have been designed to repeatedly sample during a single deployment without the need for a researcher on-site [11], [12], [13], [14]. Further, automated collection standardizes eDNA sampling and preservation, reducing the need for highly trained personnel to carry out deployment and recovery. Some automated eDNA samplers, such as the environmental sampling processor (ESP), can be mounted on autonomous underwater vehicles, allowing for biodiversity sampling over highly-resolved spatial scales in coastal-open ocean systems [11], [15]. Despite the numerous benefits of these technologies, submersible automated eDNA samplers are expensive to purchase (>100,000 USD [16]), creating a significant financial-barrier for smaller research and biomonitoring groups, and making widespread adoption impractical. Existing open-source water sampling tools [17], [18] might be adapted by the user to lower operating costs for eDNA sampling, but these require design adjustments and testing to determine effectiveness of this unintended application. Here we present the design, production, and evaluation of a purpose built open-source, low-cost (∼280 USD) subsurface automated sampler for eDNA (SASe) that filters and preserves eDNA samples *in situ*. The open-source design allows for simple modifications for user programming and design features, and the low cost of the SASe reduces the financial hurdles involved in fine-scale spatial or temporal sampling of eDNA in aquatic or marine environments.

## Hardware description

The SASe is a small (11 cm wide × 15 cm long) cost-effective (<280 USD) sampler for automated collection and preservation of aquatic eDNA. Built for field application, the SASe is submersible down to 55 m of seawater and field-programmable with an infrared remote control and menu-based interface visible on the display screen. One SASe collects a single sample at a time, using a peristaltic pump system to filter a programmable volume of water through a 0.22-µm Sterivex™ filter with a process volume of 2L (Millipore, #SVGPL10RC). Following collection, it automatically preserves the sample with DNAgard® (Biomatrica, #62001-046) [19] or similar preservative (e.g., DNA/RNA Shield from Zymo Research) [20], meaning that immediate retrieval from the field is not necessary. The SASe has two different modes of operation: 1. Daily sampling at a set time or 2. Sampling once at a set time and date. Sampling date and time are recorded on the internal microSD card for each sample. The battery life allows for deployments ranging from five days to two months, depending on the frequency and volume of sampling. The SASe can be built using tools and materials that are accessible and low cost and the construction of the majority of its components can be outsourced to companies if desired.

### Waterproof housing

**Fig. 1:**
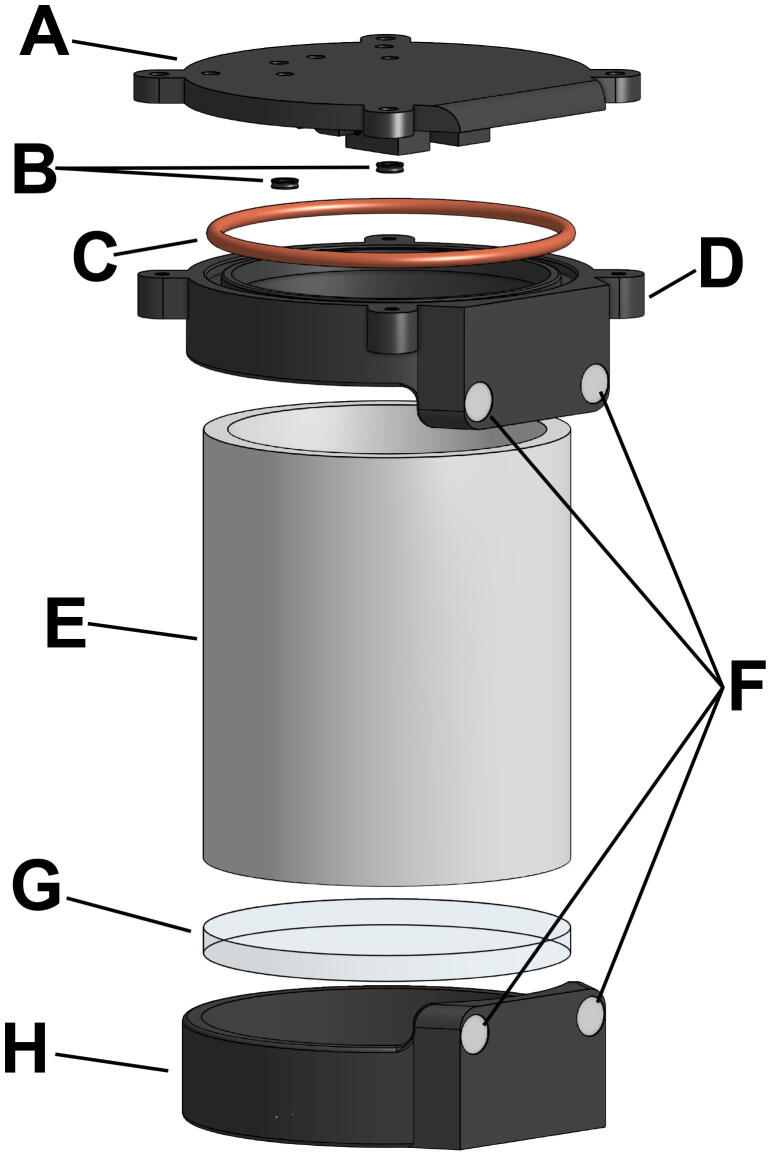
The SASe housing is made up of a 3D-printed lid (A), two X-rings for the motor shafts (B), O-ring for the lid seat (C), 3D-printed flanged end cap (D), 3″ PVC body (E), four sample cartridge connection magnets (F), ¼” acrylic window (G), and a 3D-printed fixed end cap (H).

**Fig. 2:**
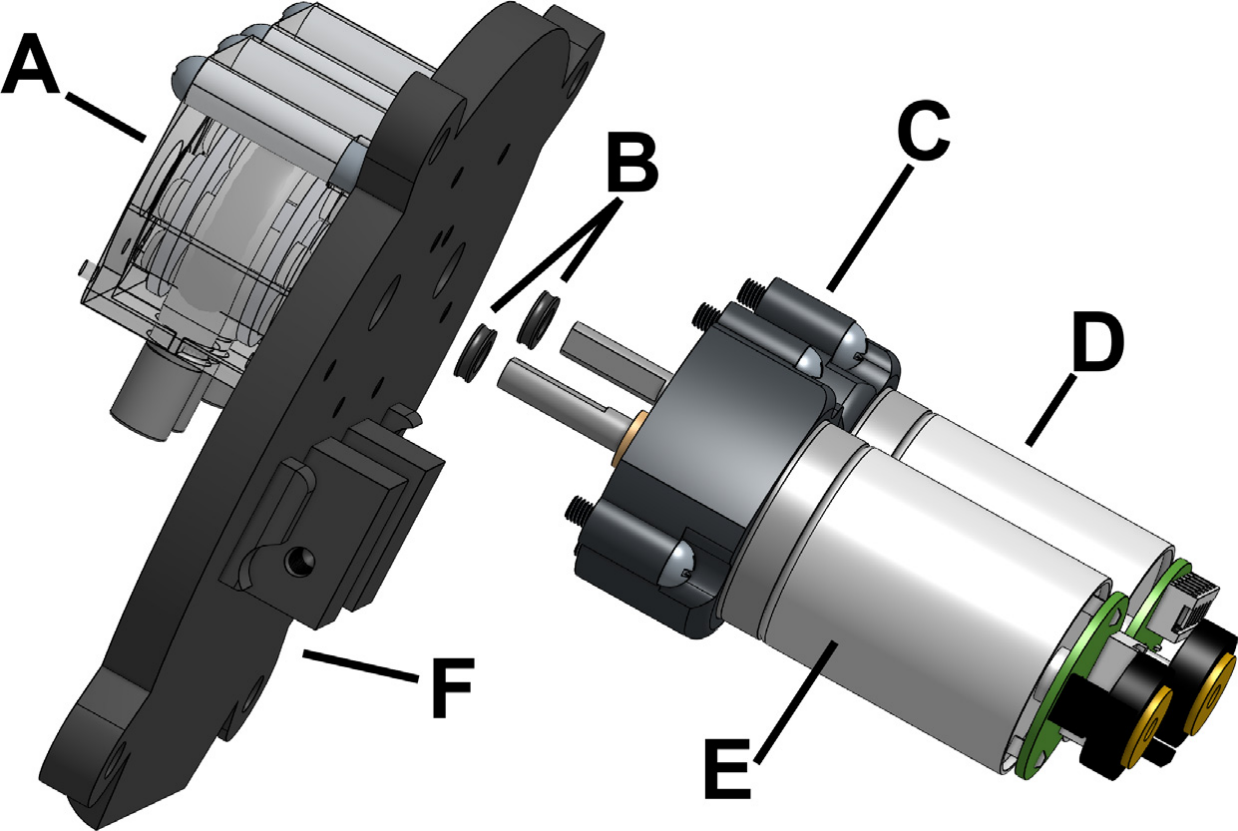
The two peristaltic pumps (A) are mounted to the outside of the 3D-printed lid (F). The 34:1 (D) and the 99:1 (E) DC motors are epoxied to 3D-printed motor sleeves (C) mounted on the inside of the lid. The motor shafts insert into the peristaltic pumps through the lid and an X-ring on the shaft of each of the motors (B) keeps the shaft-lid interface watertight.

The physical structure of the SASe was designed using the online CAD software Onshape (PTC, Boston, MA). The design utilizes a 3-inch schedule 40 PVC pipe and two 3D printed end caps, one with a 1/4-inch-thick clear acrylic window and the other with a flange and removable lid (Fig. 1). Both end caps and the acrylic window are fixed in place and made watertight with two-part epoxy. The flanged end cap connects to a removable 3D printed lid via four bolts. There are two DC gear motors mounted on the inside of the lid; the motor shafts pass through the lid and into two peristaltic pumps mounted on the outside of the lid (Fig. 2). An O-ring seated on the flanged end cap is compressed by the secured lid and seals the housing from water intrusion, while an X-ring seals the interface between each motor shaft and shaft hole. The combination of epoxy, the O-ring, and the X-rings make the SASe housing completely waterproof down to 55 m of seawater. All custom parts for the SASe were printed using desktop stereolithography (SLA) 3D printers with standard resin (Formlabs, Somerville, MA).

### Internal components

**Fig. 3:**
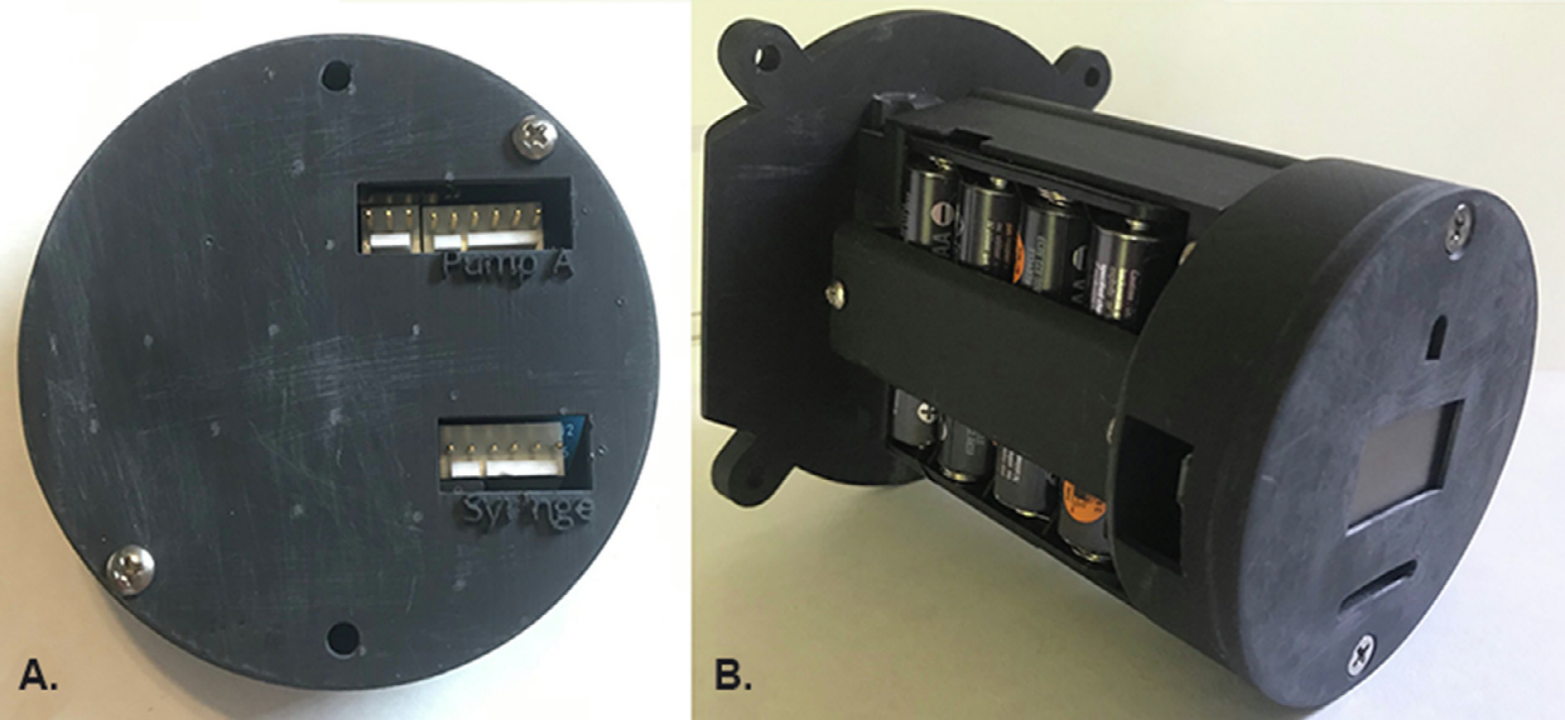
The 3D printed circuit housing (A) with openings for Molex connectors is attached to the armature (B) to connect the internal components of the SASe.

The internal components of the SASe are mounted to the inside of the lid, allowing removal from the waterproof housing in one piece when the four securing bolts are loosened (Fig. 3). The internal components include two gear motors, a 3D-printed battery housing for eight AA batteries, and a 3D-printed housing for the circuit board which includes an organic light-emitting diode (OLED) screen, an infrared (IR) sensor, and a magnetic reed switch. The DC gear motors each have an integrated 48 cycles per revolution (CPR) quadrature encoder installed for monitoring fractional rotations of the motor shafts and precisely controlling fluid transfer. The motor used for water sampling is a low-power 12 V brushed DC gear motor combined with a 34:1 metal spur gearbox, while the motor used for DNA preservative injection is a low-power 12 V brushed DC gear motor combined with a 99:1 metal spur gearbox. Both motors are mounted on the removable lid via 3D printed sleeves epoxied to the motor body (Fig. 2). Each shaft extends through the lid and is fit into one of the two OEM peristaltic pump heads (100-Series, Williamson Manufacturing Co. Ltd.) affixed to the outside of the removable lid.

### Fluid handling

**Fig. 4:**
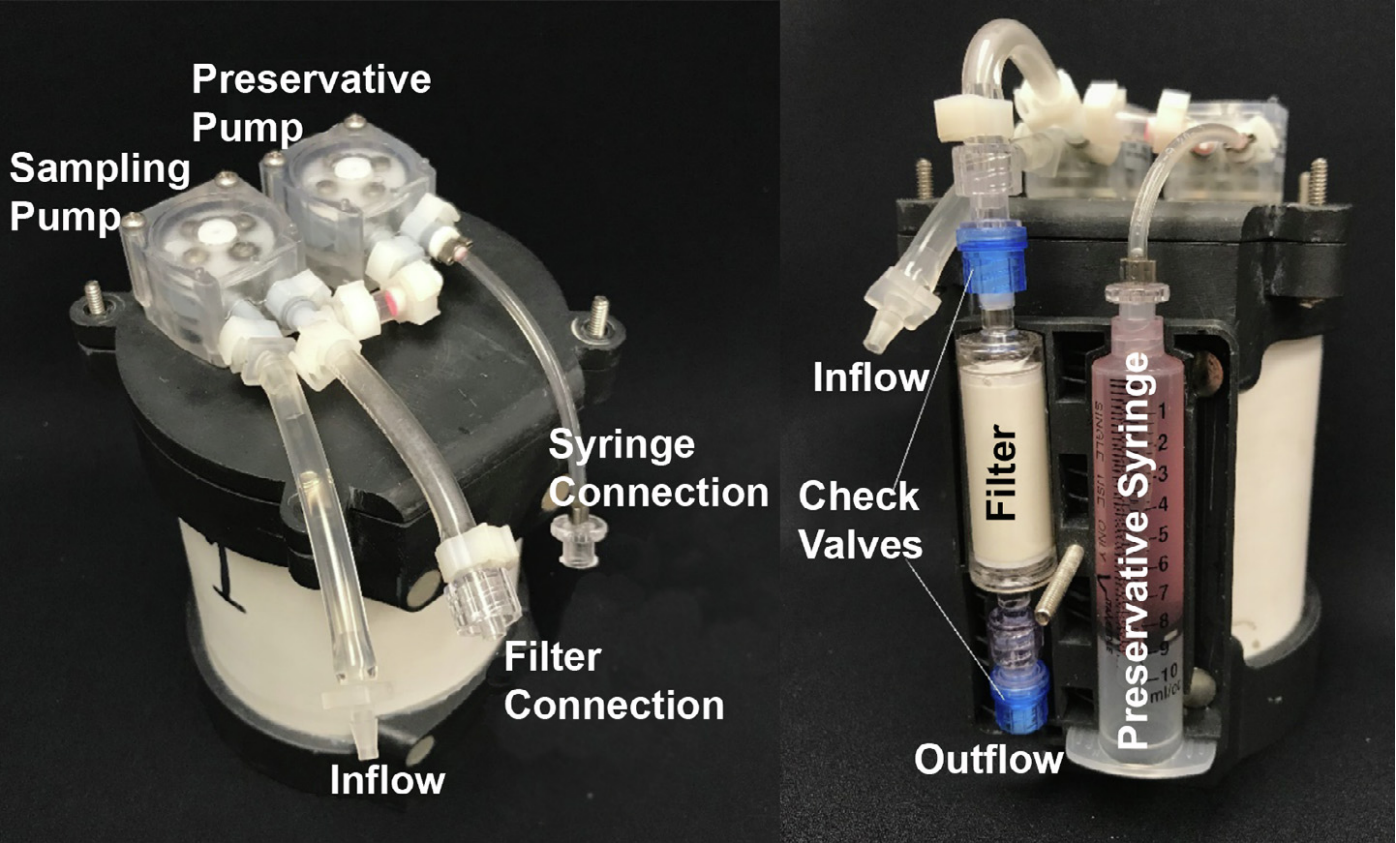
The external components of the SASe sampling system labeled for reference.

The two peristaltic pumps mounted on the SASe lid are used to collect and filter water from the surrounding environment and add preservative to the newly filtered sample. The intake of the sampling pump is open to the environment, while the intake of the preservative pump connects to a 10 mL luer lock syringe filled with DNA preservative. The outflows of both pumps are joined with a barbed tee and lead to a 0.22-μm Sterivex filter with a female luer lock inlet, upstream air vent, and male luer lock outlet. Each end of the filter has a check valve with luer lock fittings and a cracking pressure of 2.9 psi [21] to prevent dilution or contamination of the preservative and sample (Fig. 4). When sampling, a preset volume of water is pumped through the filter, followed by 5 mL of DNAgard preservative. The DNAgard purges the remaining sample water from the filter housing and saturates the eDNA on the filter to stabilize the sample at ambient environmental temperatures until recovery and extraction. The filter and preservative syringe are loaded in a 3D-printed sampling cartridge which is closed with a bolt and wing nut and magnetically affixed to the SASe housing. The modular design of the sampling cartridge and simple luer lock disconnect from the sampling tubing allows for easy exchange with fresh sampling cartridges to accommodate repeat samples.

### Electronic components

**Fig. 5:**
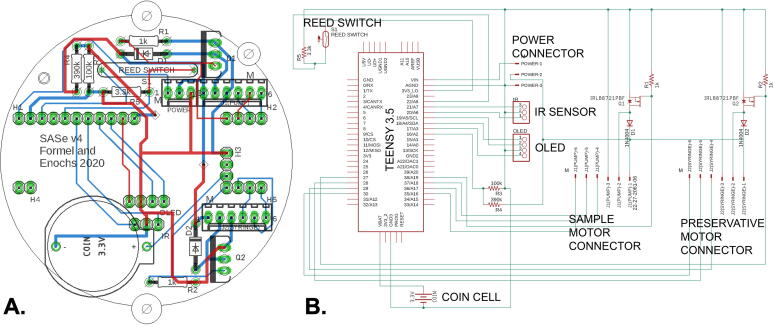
Circuit board layout (A) with pads and vias marked in green, paths in red are on the top of the board, and blue paths on the bottom. A schematic of the circuit board (B) showing connections between electronic components and connectors with major components labeled. (For interpretation of the references to colour in this figure legend, the reader is referred to the web version of this article.)

**Fig. 6:**
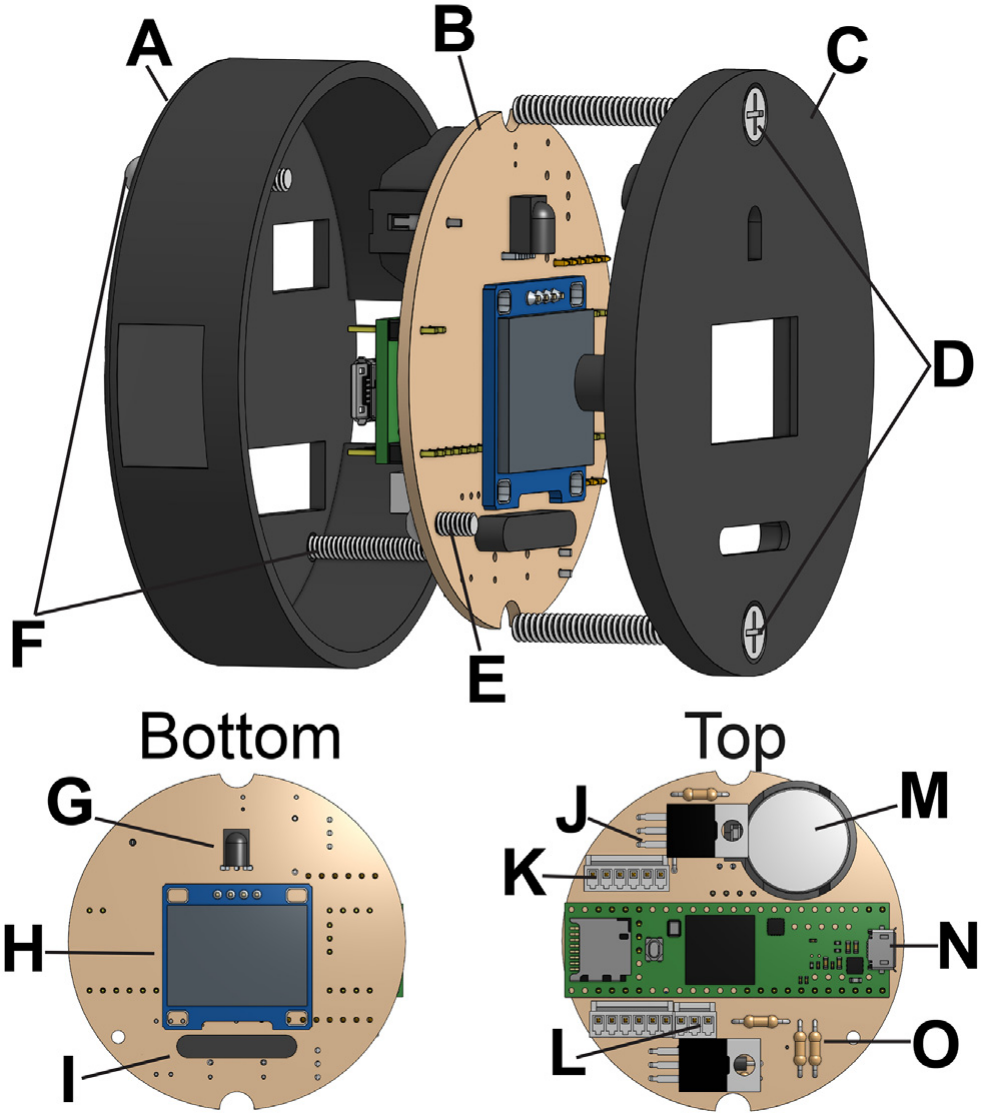
The 3D-printed circuit board housing (A) holds the SASe circuit board (B), and the UI components are visible through the 3D-printed faceplate (C). The whole housing is mounted on the main armature with two #6–32 bolts (D), while one #4–40 bolt (E) secures the circuit board to the faceplate, and two #4–40 bolts (F) hold the housing together. The circuit board is labeled to show the top and bottom and all PCB components are labeled as follows: IR receiver (G), OLED (H), reed switch (I), N-channel MOSFET (J), Molex 6-pin connector (K), Molex 3 pin connector (L), coin cell battery (M), Teensy 3.5 (N), resistors (O). Not visible are the two diodes below the MOSFETs that control current flow for the motor circuits.

The electronic hardware builds upon that of the subsurface automated water sampler (SAS) designed by Enochs et al. (2020). Hardware was designed using Autodesk Eagle (Autodesk, Inc., v 9.2.2, Fig. 5) and board prototypes were milled using two-sided copper plates and desktop circuit board milling machine (Bantam Tools, Peekskill, NY). Final circuit boards were produced by uploading Gerber files to Seeed Fusion (https://www.seeedstudio.io/fusion.html). The Teensy 3.5 microcontroller (www.pjrc.com) was selected due to its low cost, low power capabilities, built in microSD card slot, and real-time clock (RTC). The Teensy 3.5 has a 32 bit 120 Mhz ARM Cortex-M4 processor, 512 kb of flash memory, 256 kb of RAM, 58 digital I/O pins, as well as I2C pins. The user interface (UI) is visible via a 0.96-inch OLED display connected to the Teensy using the I2C communication protocol. Menu selection, sampling parameters, and manual operation are controlled using an IR remote control and a TSOP38238 IR receiver. A magnetic reed switch allows the user to wake up the SASe during low-power sleep mode. The OLED, IR receiver, and reed switch are all mounted and integrated into the underside of the circuit board (Fig. 6), making them visible through the acrylic window of the housing. The two DC gear motors are controlled using N-channel MOSFETs, and their integrated 48 CPR quadrature encoders are connected to the Teensy’s digital pins to monitor motor shaft rotation, and thereby the volumes of liquids pumped. The SASe power source is composed of eight NiMH rechargeable AA batteries wired in series in an OEM battery case. The ground, 12 V, and 6 V leads are combined into a 3-pin Molex connector. The Teensy and the UI is run with the 6 V power source, while the motors/pumps are run from the 12 V source. The battery pack voltage is measured on a Teensy analog input pin using a voltage divider. The SASe circuit board also includes a coin cell battery for maintaining the Teensy’s RTC when detached from the battery pack. The circuit board is mounted in a 3D-printed housing which has openings for easy access to both the micro USB port and the microSD card. The circuit housing is attached to the battery pack housing to connect all the internal components.

### Software

**Fig. 7:**
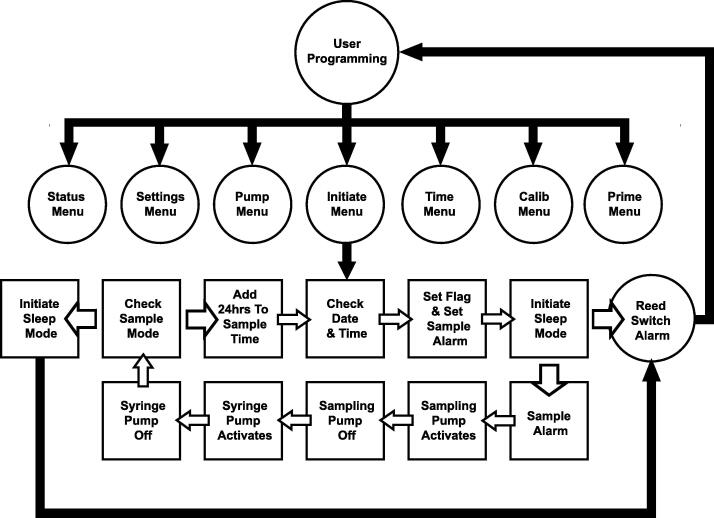
A flow chart describing the logic behind the SASe operation. The circles represent the user interface including all the menus and operation interrupt. The squares represent the automated processes. Solid arrows and lines identify areas in the programming where the user can interrupt or alter settings. Hollow arrows connecting boxes identify automation that precludes user intervention. Boxes with disconnected hollow arrows identify crossroads in the programming that depend on settings or user intervention to determine the next step.

**Fig. 8:**
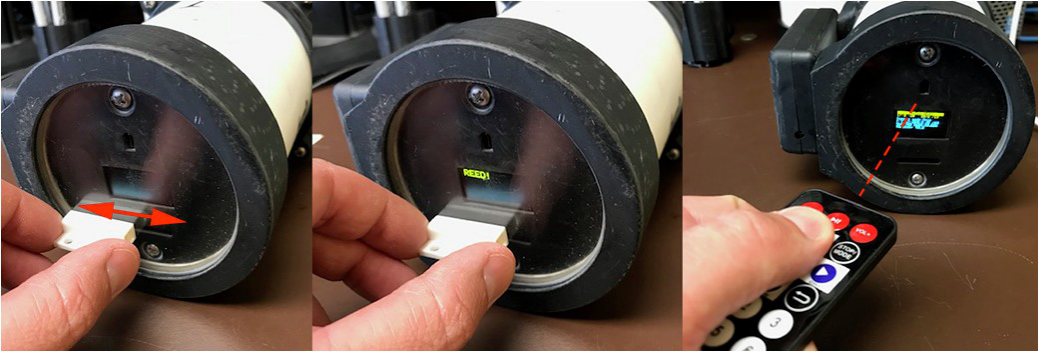
Moving a magnet across the face of the reed switch will wake the SASe from sleep mode. With line of sight, the IR remote can be used to communicate with the IR receiver for navigating through the SASe menus.

The Teensy 3.5 is USB-programmable using the freely available Arduino IDE (www.arduino.cc), with the Teensyduino add-on (www.pjrc.com). The SASe code, modified from the code for the SAS [17], relies on several freely available libraries (Table S1). The main program loop offers seven cases, covering six menu options and a sleep/operating state (Fig. 7). Navigation between menus/states is done via IR remote control (Fig. 8). IR remote programming works in day and night-time conditions, in air and underwater provided that the remote is sealed in a waterproof bag.

#### Programming

From sleep, the SASe is activated by placing a magnet next to the reed switch for > 2 s (Fig. 8). The SASe will wake-up to display the Status Menu, which includes the time, date, deployment mode, programmed collection time, volume to be collected, battery voltage, and version number for the SASe operating code. Pressing the left or right buttons on the remote control will advance the menu forward or backwards, respectively. The Settings Menu allows the user to select the sampling mode, as well as the volume of sample to be collected. The Pump Menu allows the time of collection to be entered and set. If sampling mode is set to “Daily,” the date will refer to the date when the first daily collection will be made. The Initiate Menu allows the user to begin the sampling process. The Time Set Menu is used to set the onboard real time clock. The Calib Menu allows the user to perform a calibration of the sampling pump. The Prime Menu allows the user to prime and flush the sample and preservative tubing. For each menu where selections are made, the user must select and press enter over “ENTER SET” to save the settings to the sampleParam text file on the Teensy’s microSD card. This text file is referenced by the SASe operating code and retains the most recent settings and sampling parameters. The sampleParam.TXT can also be edited through a text editing program in advance and loaded onto the microSD card. That microSD card inserted into the Teensy will functionally preprogram the SASe with the desired settings and sampling parameters.

#### Sampling

“Press Enter” in the Initiate menu begins the automated sampling process by putting the SASe in sleep mode. The code checks if pump sampling time is greater than one minute in the future, and if so, calculates the number of seconds to the programmed pumping event and puts the SASe to sleep for that amount of time. Wake-up is accomplished via an alarm at the programmed pumping time or a positive voltage on the reed switch interrupt pin. Upon wake-up, the SASe identifies which input has woken it. If it was the reed switch, it waits for half a second and confirms that the reed interrupt pin is high, two times. If the reed input is sustained, the SASe remains awake and defaults to the Status menu. If not, the SASe assumes that it was an erroneous wakeup and reinitiates the sleep/initiate process. If the wake-up input is not the reed pin (i.e., the RTC alarm), the SASe checks the alarm flag for the pump, executing the following logic for each independently. If the alarm flag is zero and the time is greater than the alarm time, the alarm is set to one. If the alarm flag is one, the sampling pump is turned on, the time at which sampling should be finished is calculated, data is logged, and the alarm flag progresses to two. Sampling continues until the time is greater than the sample end time, at which point the sampling pump turns off, the preservative pump turns on, and the alarm flag is set to three. If the alarm flag is three, the time at which pumping should be finished is calculated, and the pump continues to run until the time is greater than the pumping end time, at which point the preservative pump turns off and the sampling mode is identified. If the sampling mode is only a single collection time, then the alarm flag is set to four. If daily sampling is selected, the alarm flag is set back to zero and the alarm time is updated with an additional 86,400 s (24 hrs). Upon updating the alarm flags and sample time, the SASe returns to sleep until “woken up” by the next alarm.

• Open source eDNA sampler for collecting and preserving one sample at a time
• DNAgard preserves the eDNA sample at ambient conditions.
• Inexpensive design (<280 USD) allows production of replicate samplers for fine-scale temporal and spatial sampling.
• Waterproof to 55 m and can be programmed in the field.

## Design files

**Table:** 

Design file name	File type	Open source license	Location of the file
SASeV3c.ino	Arduino code	CC BY 4.0	Source file repository
SASeBoard15.zip	Gerber Files	CC BY 4.0	Source file repository
SASe3DPrintFiles.zip	3Dprint Files	CC BY 4.0	Source file repository
SASeVectorFiles.zip	Vector Files	CC BY 4.0	Source file repository
BillofMaterials.docx	MS Word Doc	CC BY 4.0	Source file repository
BuildInstructions.pdf	PDF	CC BY 4.0	Source file repository
OperationInstructions.pdf	PDF	CC BY 4.0	Source file repository

**SASev3c.ino** is the Arduino code for the SASe programming and operation.

**SASeBoard15.zip** is a zipped folder containing the Gerber files for milling or ordering the SASe circuit board.

**3DprintFiles.zip** is a zipped folder containing all the stereolithography CAD (STL) files for 3D printing the components of the SASe.

**SASeVectorFiles.zip** is a zipped folder containing the vector files for laser cutting, milling, or ordering the acrylic window and test cap for the SASe.

**BillofMaterials.docx** is a Microsoft Word document for the detailed bill of materials.

**BuildInstructions.pdf** is a pdf document for standalone use as a construction manual for the SASe.

**OperationInstructions.pdf** is a pdf document for standalone use as an operations manual for the SASe.

## Bill of materials

The detailed bill of materials is included as a supplemental word document in the source file repository at https://doi.org/10.17632/y3dgh36zt9.2. While the SASe was designed and constructed with both function and cost in mind, federal purchasing guidelines limited sources for SASe components. The parts and associated sources in the bill of materials here represent what was used in the development and construction of the SASe, however, it is likely that open access to all retailers and distributors would allow for lower overall parts cost to build the SASe.

## Build instructions

Detailed build instructions are included as a supplemental pdf in the source file repository at https://doi.org/10.17632/y3dgh36zt9.2 to be used as a standalone reference for construction of the SASe. While the SASe is meant to embrace simplicity in its design, some basic builder and circuitry skills are needed, including familiarity with 3D printing and soldering. The SASe design files can be used to order the custom parts from online businesses to reduce the in-house labor involved in building the SASe, however, this will increase the cost of construction.

## Operation instructions

The detailed operation instructions, including user interface, setup, deployment, and sample recovery, are included as a supplemental pdf in the source file repository at https://doi.org/10.17632/y3dgh36zt9.2 to be used as a standalone reference for operation of the SASe.

## Validation and characterization

Several tests, guided by the validation methods of Enochs et al. 2020, were conducted in the lab to evaluate the performance and limitations of the SASe.

### Housing integrity

To assess the waterproof nature of the SASe housing, units were submerged in water within a pressure chamber and repeatedly brought to 80 psi, equivalent to 55 m of seawater. Pressure was increased and decreased in the chamber at a rate of 12–18 m per minute. SASe’s were exposed to pressurized conditions in both sleep and sampling modes to test the water-tight seal of the X-rings on the motor shafts. Three SASe’s were also kept at depth for multiple days to test the longevity of the seal. The SASe housings performed at depth, with no water intrusion detected. It is worth noting that while the SASe design was tested under conditions equivalent to 55 m depth in seawater, this limit was imposed by the capabilities of the pressure chamber and does not necessarily represent the maximum waterproof depth limit of the SASe housing. Deeper testing is required to determine the actual depth limit of the housing.

### Sample volume accuracy

**Fig. 9:**
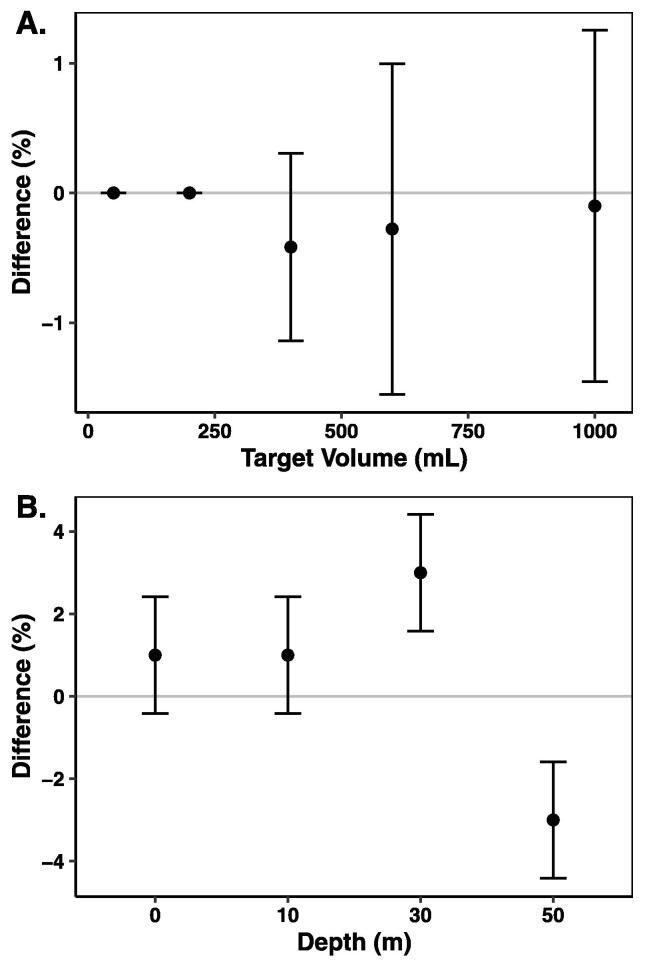
Percent difference in the volume of water programmed to be filtered vs that collected as a function of (A) targeted volume and (B) depth. Error bars are standard deviations.

The accuracy and precision of the volume of water filtered by the SASe was evaluated by calibration of three separate SASe samplers, which were each subsequently programmed to filter five different volumes of water (50, 200, 400, 600, and 1000 mL) in random order. The samplers were run on a benchtop and tubing was added to connect the inflow of the sampling pump to a water source and to route the outflow to a graduated cylinder, both sets of tubing were primed prior to testing. The resulting volumes of water filtered were measured in the graduated cylinder and compared to the set volumes. The standard deviation of the sampling volumes was ± 0.01 per target mL (Fig. 9A), meaning a 1000 mL sample could actually be 1000 mL ± 10 mL. The rotary encoders measure motor shaft rotations (i.e., pump rotations) down to a fraction of a rotation, but there is slight variation in pumping efficiency with the peristaltic pumps that can lead to volume error. As pumping volume increases and the filter becomes more clogged, the backpressure in the system can also alter the pumping efficiency. For this reason, smaller sample volumes should be set in highly turbid water to avoid unexpected reduction in actual sample volume. While this is fine for coastal environments, where biomass is high, in open ocean systems with lower biomass the need for large sample volumes for detection of eDNA could require an alternate filter size or greater replication of samples.

### Sample volume at depth

The potential for pressure to impact sample volume was evaluated using the same pressure chamber and two SASe samplers programmed to collect 250 mL through a filter and into a sample bag. The collections were performed at 0, 15, 44, and 73 psi (comparable to 0, 10, 30, 50 m depth in seawater, respectively) in random order. The volume of each collected sample was subsequently measured using a graduated cylinder. The sampling volumes for the two samplers were in the range of variability expected by the SASe system at the surface and 10 m depth (250 mL ± 2.5). At 30 and 50 m depth, the variability from the target volume increased to 250 mL ± 7.5. The increased variability implies some effect from depth on the target volume, but, similar to findings by Enochs et al. (2020), the relationship between depth and volume pumped was inconsistent (Fig. 9B) and of insufficient magnitude to warrant further design alterations.

### Sample path integrity

In order to test the watertight seal of the SASe sampling system and ensure no dilution or contamination of the sample or preservative would occur, twelve Sterivex filters were capped with luer lock check valves, identical to the configuration on the SASe’s. All the filters were filled with a solution of dilute Rhodamine B (0.04% Fluorescent Red Dye) and exposed to a dynamic treatment (i.e., vigorous mixing), with half of the filters in the air and half underwater to simulate the conditions of a deployment. The filters were then emptied into cuvettes and absorbance of the filtrate was measured in a HACH DR6000 spectrophotometer (Accuracy ± 5 mAbs) [22]. Absorbance of test filters were compared to a dilution curve of the dye solution. A control treatment consisting of a cuvette filled directly with the dilute Rhodamine B solution was also run through the spectrophotometer for reference.

**Fig. 10:**
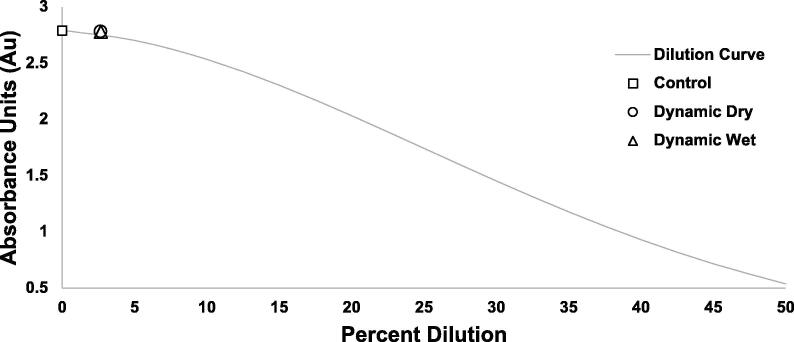
The average absorbance units measured for the wet and dry filter treatments and the control graphed on the dilution curve for a dilute Rhodamine B solution.

The dilution test demonstrated the SASe sampling system is watertight (Fig. 10). Results showed absorbance unit (Au) values of 2.793 (Std Dev ± 0.039) for the control treatment, 2.645Au (Std Dev ± 0.031) for the dry dynamic treatment, and 2.667Au (Std Dev ± 0.044) for the wet dynamic treatment. While the treatment values are lower than the control, the similarity of the wet and dry treatments indicate the difference is due to absorption of the dye by the filter media and not infiltration and dilution by water.

### Power consumption

**Fig. 11:**
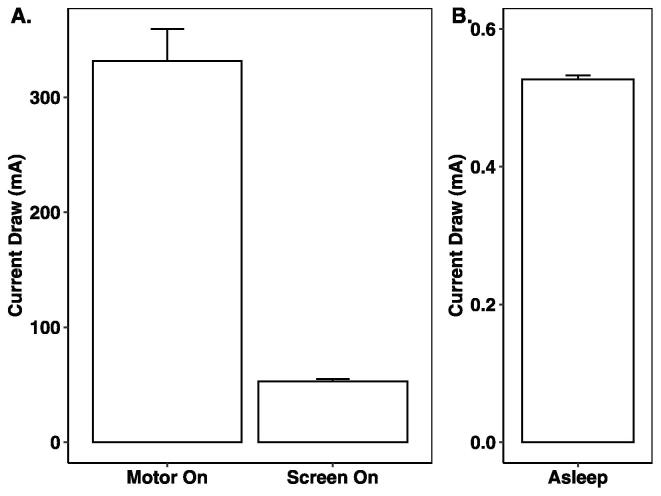
Current consumption during three operational states: (1) Motor On, while the pump is sampling water, (2) Screen On, while the OLED screen is on and receiving signals from an infrared remote control, and (3) Asleep, while the SASe is dormant, waiting until the next sampling event. Current draw was measured across the four cells powering the microcontroller and electronic components, as well as all eight cells used to power the motors while sampling. Error bars are standard deviations.

The current draw and battery longevity were tested two separate ways. First, three SASe samplers were filled with eight fully charged NiMH AA batteries (1.2 V, 2450 mAh, Eneloop pro, Panasonic) and the current draw was measured using a handheld multimeter (EX330, Extech Instruments) during sleep mode, programming mode with the screen on, and during sampling following the method of Enochs et al. (2020). To compare the battery life with real-world battery life, three SASe’s with fresh batteries were programmed with an edited code to sample 1000 mL every one hour and the battery voltage was logged before and after each sampling. All pumping was done under load, with water circulating through the sampling system. In sleep mode, the SASe draws very little current at 0.53 mA ± 0.005 mA (Fig. 11). When the OLED screen is on, the current draw on the SASe increases to 52.88 mA ± 1.62 mA. The highest current draw occurs while the motors are running for sampling, at 331.45 mA ± 22.81 mA. Given these results and the size of the batteries (8 × 2450 mAh), conservatively calculating the lifespan as half the batteries’ charge, the SASe can remain asleep for up to 96 days, awake for ∼ 1 day, or running for 3.6 hrs before fully discharging its batteries. The deployed SASe will take at least one sample during deployment, taking approximately 35 min to collect one sample, reducing the battery life to 81 days. If the SASe is running in daily mode, the total battery life will be 5.9 days.

**Fig. 12:**
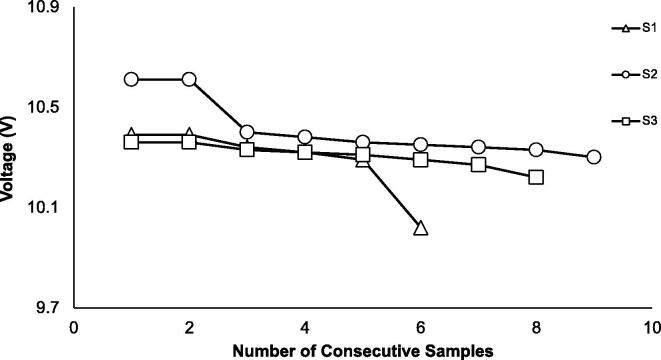
Voltage measurements in three SASe set to collect 1000 mL hourly until battery charge became insufficient to power SASe. Count of complete sampling cycles do not include the last sampling event.

The real-world assessment of battery life for three SASe’s exhibited a lifespan of five to eight complete sampling cycles (Fig. 12), correlating well with the theoretical assessment based on current measurements. The largest current draw requirement occurs during the sampling period, while current draw during other periods (e.g., sleep time) are comparatively small. This real-world test was done hourly but translates well to a multi-day sampling event due to the negligible current draw during sleep mode. The range of sampling events completed, as well as the variable starting voltages of each SASe, reinforces the need to properly charge batteries in advance of deployment to insure maximum deployment and sampling capability.

### Efficacy of DNA recovery

To test the impact of filtration (SASe vs. manual pump) on eDNA recovery, two similar tests were run to compare DNA yield. The first test was done using a seawater solution inoculated with *Enterococcus faecalis* bacteria to boost available eDNA in the sample water, and the second test was done using unaltered seawater collected from a nearby dock. Both tests employed a manual desktop peristaltic pump and replicate SASe samplers to compare filtration methods using the same water source.

#### Test 1: eDNA enhanced seawater

For the first test, 20 L of coastal seawater was collected in a sterile carboy from Bear Cut Virginia Key, Florida (25.731667, −80.161667). In the laboratory, the volume of the seawater sample in the carboy was aseptically measured and an aliquot from a previously prepared viable *Enterococcus faecalis* positive control stock culture was spiked into the seawater sample to bring the final known concentration of enterococci cells in the carboy to 1x10^5^ colony forming units per liter. The spiked seawater was mixed by vigorous agitation to ensure a homogenous solution. Three SASe’s were set to filter 1 L of the spiked sample three times, each in serial fashion with a fresh Sterivex filter per liter for a total of nine samples. A manually controlled peristaltic pump (Masterflex, Cole-Parmer) was used for the manual sampling method to filter 1 L of the spiked solution through each of five independent Sterivex filter cartridges (of the same model) in serial fashion. For both methods, seawater was pulled from the carboy using silicone intake tubing and the filtrate was collected to determine actual volume filtered. Prior to sample filtration all equipment, including SASe and peristaltic pump tubing, was cleaned using 10% HCl solution and distilled water, followed by multiple rinses in molecular-grade water. The SASe automatically filled each Sterivex with DNAgard preservative solution after filtration, while a cleaned syringe was used to manually fill each Sterivex from the manual method with DNAgard preservative following filtration. All Sterivex cartridges were sealed with luer lock plugs and stored at room temperature for one week prior to extraction and analysis. For this test 10% HCl was used to pre-clean the SASe sampling system, however, later tests and the final cleaning protocol use a dilute bleach solution for user convenience. Both methods are used for cleaning of eDNA equipment prior to sampling [3], [4].

Extraction methods for Sterivex filters were the same for each treatment. DNAgard preservative was drained from the filter cartridge under gentle positive pressure using a sterile luer-tip syringe. The Sterivex cartridge capsule was aseptically cut open using an ethanol sanitized PVC pipe cutter, the inner core of the cartridge containing the filter membrane removed, and the filter membrane was aseptically cut into pieces and placed into a “lysing matrix E” bead beat tube (MPBiomedicals). Total metagenomic eDNA was then extracted by lysing and homogenizing the cells collected on the filter in the lysing matrix E tubes using the FastDNA Spin Kit for Soil (MPBiomedicals). Manufacturer’s directions were followed, using a FastPrep-24 bead-beating homogenizer instrument with a speed setting of 6.0 m/s for 60 s each, and finally eluting the purified DNA from the binding resin into a final volume of 100 µL. Total DNA yield from each extraction was measured by fluorometry on a Qubit 3 fluorometer (Thermo Fisher), using the Quant-iT HS dsDNA Quantitation Kit (Thermo Fisher) as per manufacturer instructions.

**Fig. 13:**
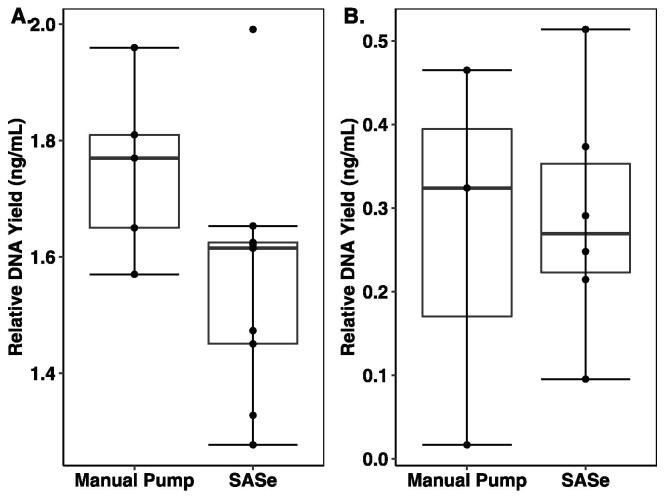
A comparison of the relative DNA yield for the standard sampling method compared with the SASe based on the volume filtered (milliliters) and total DNA yield (nanograms) from the Qubit measurements for the eDNA enhanced seawater test (A) and for the unaltered seawater test (B). Errors bars represent deviation from the mean and the points are replicate samples. The difference in relative DNA yield between sample methods for both tests was not significant (P = 0.0998, P = 0.8693 respectively).

DNA yield for each extraction was standardized by the volume of spiked seawater solution filtered and calculated as the average yield of nanograms of DNA per milliliter (ng/ml) of sample for each filtration method (Fig. 13A). Differences in relative DNA yield for the two sample methods was evaluated using an unpaired two sample *t*-test and found to be not significant (*t*(12) = 1.7835, *p* = 0.0998) with averages of 1.5594 ng/mL ± 0.2120 (n = 9) and 1.7520 ng/mL ± 0.1504 (n = 5) for the manual pump and SASe respectively.

#### Test 2: Unaltered seawater

For the second test, 20 L of coastal seawater was collected in a sterile carboy from Bear Cut and immediately transferred to the lab for filtration. Six SASe were set to filter 1 L of the seawater through Sterivex filters for a total of six samples while a manually controlled Masterflex peristaltic pump was used to simultaneously filter 1 L of seawater through a total of six Sterivex filters (same model). For both methods, seawater was pulled from the stock carboy using silicone intake tubing, the collected seawater was gently homogenized throughout filtration, and the filtrate from each filter was collected and measured in a graduated cylinder to verify volume filtered. All equipment, including SASe and peristaltic pump tubing, was cleaned in advance using a 5% bleach solution in distilled water, followed by multiple rinses in molecular-grade water. SASe filters were automatically filled with DNAgard preservative, while the manual method required the Sterivex be filled with preservative using a cleaned syringe. All Sterivex filters were capped with luer caps and stored at room temperature until DNA extraction, which was performed within two months.

Modifications to an intra-cartridge bead beating protocol [23] were used to extract DNA from Sterivex filters. Approximately half of the DNAgard (∼1 mL) was filtered out of the Sterivex using a syringe with gentle pressure. Samples were processed using the ZymoBIOMICS 96 MagBead DNA kit following the manufacturer’s instructions, with the exception of bead beating, which was performed within the Sterivex cartridge filters. Premade Zymo bead mixtures (0.1 mm silica + 0.5 mm zirconium) were directly added into the cartridge, followed by vortexing (via Vortex Genie) for 40 min. DNA lysates were transferred to 1.2 mL lo-bind tubes using a syringe and centrifuged at 10,000×g. Supernatant (∼600 µL) was transferred to 96-well KingFisher plates and split across 3 wells (200 µL × 3 per sample); MagBinding buffer (600 µL) and beads (25 µL) were added to each individual well. Samples were extracted using an automated KingFisher Flex instrument (Thermo Fisher) with a custom-made script, which included the sample plate (with binding buffer and beads added), three plates for washing (500–900 µL per well), and one plate for DNA elution (50 µL per well) in DNase/RNase free water. Following the KingFisher run, sample wells were recombined, yielding ∼150 µL of eluted DNA. Total DNA yield was measured on a Qubit 3 fluorometer (Thermo Fisher), using a Qubit dsDNA HS kit (Thermo Fisher).

DNA yield for each extraction was standardized by the volume of spiked seawater solution filtered and calculated as the average yield of nanograms of DNA per milliliter (ng/ml) of sample for each filtration method (Fig. 13B). Differences in relative DNA yield for the two sample methods was evaluated using an unpaired two sample *t*-test and found to be not significant (*t*(7) = −0.17072, *p* = 0.8693) with averages of 0.2685 ng/mL ± 0.2292 (n = 3) and 0.2893 ng/mL ± 0.1431(n = 6) for the manual pump and SASe respectively.

The outcome of the two DNA validation tests showed similar DNA capture for the SASe and manual peristaltic pump, demonstrating the effectiveness of the SASe automated system for capturing and preserving eDNA for downstream analysis and presence/absence detection of aquatic organisms.

## Summary

Capabilities of the SASe

● Collects and preserves one eDNA sample per filter at ambient environmental temperature until sample can be retrieved.
● Sample recovery and analysis allows for presence/absence detection.
● Replicate SASes can increase temporal and spatial sampling capabilities.
● Capable of filtering up to two liters per filter with pump volume accuracy ±3% of target volume after calibration.
● Waterproof to 55 m.
● Remote programming works in the field.

## Declaration of Competing Interest

The authors declare that they have no known competing financial interests or personal relationships that could have appeared to influence the work reported in this paper.
