# Supplementary material for: Subsurface automated samplers for eDNA (SASe) for biological monitoring and research
Source: HardwareX. 2021 Oct 14;10:e00239. doi: 10.1016/j.ohx.2021.e00239 (PMC9123479; doi:10.1016/j.ohx.2021.e00239)
Supplement: Supplementary data 1 [file mmc1.docx]

| **Library** | **Purpose** | **Author** | **Source** |
| --- | --- | --- | --- |
| TimeLib.h | Time functions | Stoffregen, P | https://github.com/PaulStoffregen/Time/blob/master/TimeLib.h |
| Wire.h | I2C communication | Zambetti, N; Krein, T | https://github.com/esp8266/Arduino/blob/master/libraries/Wire/Wire.h |
| Snooze.h | Teensy sleep and low power function | Duffy, C | https://github.com/duff2013/Snooze |
| Adafruit_GFX.h | Adafruit OLED display | Fried, L; Adafruit | https://github.com/adafruit/Adafruit-GFX-Library |
| Adafruit_SSD1306.h | Adafruit OLED display | Fried, L; Adafruit | https://github.com/adafruit/Adafruit_SSD1306 |
| IRremote.h | IR communication | Shirriff, K; Knapp, J; Lauszus, K., et al. | https://github.com/z3t0/Arduino-IRremote |
| SdFat.h | microSD card write and read functionality | Greiman, B | https://github.com/greiman/SdFat |
| SPI.h | Serial communication | Maglie, C; Stoffregen, P; Kooijman, M; et al. | https://github.com/PaulStoffregen/SPI/blob/master/SPI.h |
| ADC.h | Analog to digital converter | Villanueva, P | https://github.com/pedvide/ADC/blob/master/ADC.h |
| Encoder.h | Quadrature encoder functions | Stoffregen, P | https://github.com/adafruit/Adafruit_MCP9808_Library/blob/master/Adafruit_MCP9808.h |

Supplemental Table 1: A list of the libraries used in the SASe code, each labeled with its purposes, author, and GitHub URL
